# Plasma alterations in immunoglobulin G, immunoglobulin M, immunoglobulin A, serum transferrin, serum albumin, prealbumin, interleukin 6 and serum C-reactive protein after immune-type enteral nutrition support in patients undergoing radical resection of colon cancer

**DOI:** 10.5937/jomb0-55416

**Published:** 2025-10-28

**Authors:** Xiaoxu Cui, Haiping Wu

**Affiliations:** 1 The First Affiliated Hospital of Soochow University, Department of General Surgery, Soochow, Jiangsu, China; 2 The First Affiliated Hospital of Soochow University, Hemodialysis Center, Soochow, Jiangsu, China

**Keywords:** colon cancer, radical resection, high-quality nursing, immune, enteral nutrition, plasma alterations in immunoglobulin G (IgG), immunoglobulin M (IgM), immunoglobulin A (IgA), serum transferrin (TFN), serum albumin (ALB), prealbumin (PA), interleukin (IL-6), serum C-reactive protein (CRP), karcinom kolona, radikalna resekcija, visoko kvalitetna nega, imunitet, enteralna ishrana, promene u plazmi imunoglobulina G (IgG), imunoglobulina M (IgM), imunoglobulina A (IgA), serumskog transferina (TFN), serumskog albumina (ALB), prealbumina (PA), interleukina 6 (IL-6), serumskog C-reaktivnog proteina (CRP)

## Abstract

**Background:**

This study aims to evaluate the effects of high-quality nursing combined with immune-type enteral nutrition (IEN) support on postoperative recovery, nutritional status, immune function, inflammatory response, and complication rates in patients undergoing radical colon cancer resection.

**Methods:**

A total of 106 patients with colon cancer who underwent radical resection were randomly divided into a control group (CG) and an observation group (OG). The CG received routine nursing care and parenteral nutrition support, while the OG received high-quality nursing care and immune-type enteral nutrition support. Key outcomes were assessed, including recovery times, nutritional markers, immune function, inflammatory response, quality of life (QLQ-C30 scores), complication incidence, and nursing satisfaction.

**Results:**

The OG demonstrated significantly shorter recovery times for bowel sounds, exhaust gas, defecation, time to get out of bed, and overall hospital stay compared to the CG (P&lt;0.05). The incidence of complications was also lower in the OG (3.77% vs. 16.98%, P=0.025). Nutritional markers such as serum transferrin (TFN), prealbumin (PA), and albumin (ALB) were significantly higher in the OG (P&lt;0.05), along with increased levels of immunoglobulins (IgG, IgA, and IgM) and reduced inflammatory markers (CRP and IL-6, P&lt;0.05). Quality of life scores and nursing satisfaction were significantly better in the OG (P&lt;0.05).

**Conclusions:**

High-quality nursing combined with immune-type enteral nutrition support significantly enhances postoperative recovery, improves nutritional and immune status, reduces inflammation, lowers complication rates, and boosts the quality of life and nursing satisfaction in patients undergoing radical resection for colon cancer. This approach provides an effective strategy for promoting better outcomes in this patient population.

## Abstract (sr)

**Uvod:**

&nbsp;Ova studija ima za cilj da proceni efekte visokokvalitetne nege u kombinaciji sa imunološkom enteralnom nutritivnom (IEN) podrškom na postoperativni oporavak, nutritivni status, imunološku funkciju, inflamatorni odgovor i učestalost komplikacija kod pacijenata podvrgnutih radikalnoj resekciji karcinoma kolona.

**Metode:**

Ukupno 106 pacijenata sa karcinomom kolona, koji su bili podvrgnuti radikalnoj resekciji, nasumično je podeljeno u kontrolnu grupu (CG) i grupu posmatranja (PP). CG je primala standardnu negu i parenteralnu nutritivnu podršku, dok je OG, pored visokokvalitetne nege, dobijala i imunološku enteralnu nutritivnu podršku. Procenjivani su ključni ishodi, uključujući vreme oporavka, nutritivne pokazatelje, imunološku funkciju, inflamatorni odgovor, kvalitet života (QLQ-C30 skor), učestalost komplikacija i zadovoljstvo negom.

**Rezultati:**

OG je imala značajno kraće vreme oporavka u pogledu ponovne pojave crevnih zvukova, ispuštanja gasova, defekacije, vremena ustajanja iz kreveta i ukupne dužine boravka u bolnici u poređenju sa CG (P&lt;0,05). Učestalost komplikacija bila je niža u OG (3,77% u odnosu na 16,98%, P=0,025). Nutritivni pokazatelji, poput serumskog transferina (TFN), prealbumina (PA) i albumina (ALB), bili su značajno viši u OG (P&lt;0,05), uz povećane nivoe imunoglobulina (IgG, IgA i IgM) i smanjenje inflamatornih markera (CRP i IL-6, P&lt;0,05). Skor kvaliteta života i zadovoljstvo negom bili su značajno bolji u OG (P&lt;0,05).

**Zaključak:**

Visokokvalitetna nega u kombinaciji sa imunološkom enteralnom nutritivnom podrškom značajno poboljšava postoperativni oporavak, nutritivni i imunološki status, smanjuje inflamaciju, učestalost komplikacija i poboljšava kvalitet života i zadovoljstvo negom kod pacijenata pod-vrgnutih radikalnoj resekciji karcinoma kolona. Ovaj pristup predstavlja efikasnu strategiju za postizanje boljih ishoda u ovoj populaciji pacijenata.

## Introduction

Colon cancer ranks third in the incidence of various gastrointestinal malignancies, which shows that its incidence is very high [1]. In recent years, with the transformation of Chinese residents’ diet structure to high-fat and high-calorie, the incidence of colon cancer has risen significantly, and relevant statistics show that there are about 370,000 new cases of colon cancer in China every year [2]. Although the cause of bowel cancer is not yet clear, possible factors include medical history, family history, and poor diet [3]. In terms of medical history, early inflammatory colorectal adenoma and adenomatous polyps may lead to colon cancer [4]. In terms of family history, patients with a family history of colon cancer are more likely to develop the disease [5]. In terms of eating habits, if the patient consumes too much fat or protein, or the intake of dietary fibre can not meet the body’s needs for a long time, it also increases the incidence of colon cancer [6].

The postoperative recovery of patients undergoing radical resection of colon cancer is often hindered by various factors, including malnutrition, compromised immune function, and increased inflammation. Effective nutritional support is crucial in enhancing recovery outcomes and improving the overall prognosis. Recent studies have highlighted the importance of immune-enhanced enteral nutrition in mitigating these challenges by improving nutritional status, boosting immune function, and reducing inflammation [7]. Specifically, immune-type enteral nutrition, rich in key nutrients such as glutamine, arginine, and omega-3 fatty acids, has shown promise in supporting the recovery of gastrointestinal function, modulating immune responses, and reducing inflammatory markers. This study investigates the impact of immune-type enteral nutrition on key plasma and serum biomarkers – namely immunoglobulins (IgG, IgM, and IgA), nutritional proteins (transferrin, albumin, and prealbumin), and inflammatory markers (IL-6 and C-reactive protein) – in patients undergoing radical colon cancer resection, providing valuable insights into its effects on immune modulation and nutritional recovery [8] [9].

After the onset of the disease, the symptoms of the patient are dyspepsia, abdominal distension, mucous stool, bloody stool, etc., later may appear lower digestive tract bleeding and intestinal obstruction and other complications; cancer cells can also be transferred to the liver and other organs, which has a greater impact on the health of the patient [10]. The main clinical treatment of colon cancer is surgical resection. Still, the patients are prone to a variety of complications after surgery, and the degree of gastrointestinal dysfunction is also different [11]. Therefore, it is necessary to implement scientific nursing intervention, improve patients’ postoperative gastrointestinal function, further promote their rehabilitation, and effectively shorten their hospitalisation time.

High-quality nursing is a commonly used nursing method in clinics in recent years, which can reduce the psychological pressure caused by disease, surgical stimulation, and postoperative breast appearance change [12]. At the same time, high-quality nursing can also improve patients’ compliance, which is of great help in promoting surgical safety [13].

In the continuous proliferation of malignant tumour cells, a large amount of energy needs to be consumed, resulting in increased basic metabolism of patients and accelerated protein decomposition [14]. At the same time, surgical treatment further enhances the catabolism of patients. Therefore, patients with colon cancer are often accompanied by different degrees of malnutrition and decreased immune defence ability after surgery [15]. Enteral nutrition is widely used in the postoperative nutritional support treatment of patients with gastrointestinal tumours because it is more in line with human physiological characteristics and has less impact on the function of the liver, kidney and other important organs than parenteral nutrition [16]. Conventional enteral nutrition is insufficient to improve patients’ postoperative immune defence function. Tumour nutrition proposes that adding specific nutrients to enteral nutrition can enhance the patient’s immune response [17].

Our study aimed to explore the impact of high-quality nursing combined with immune-type enteral nutrition support on postoperative recovery and nutritional status in patients undergoing radical resection of colon cancer.

## Materials and methods

### General data

From January 2023 to December 2024, 106 patients with colon cancer who underwent radical resection of colon cancer were selected as the study objects. Inclusion criteria: (1) CT and pathological examination confirmed colon cancer; (2) In line with the indications of radical resection of colon cancer; (3) No history of neoplastic disease. Exclusion criteria: (1) Mental abnormality, inability to complete the study; (2) History of chemotherapy or radiotherapy; (3) Complicated with cardiovascular and cerebrovascular diseases and malignant tumours in other parts. The patients and their families understood the content of this study and voluntarily signed the informed consent form. Our hospital’s Medical Ethics Committee approved the study. Patients were divided into a control group (CG) and an observation group (OG) according to the random number table method, with 53 cases, respectively. There were 28 males and 25 females in the CG. The average age was (50.26±5.25), ranging from 24 to 79 years. Clinical stages: Stage I (12 cases), stage II (22 cases), stage III (19 cases); Tumor location: descending colon (16 cases), transverse colon (18 cases), ascending colon (19 cases). There were 30 males and 23 females in the CG. The average age was (50.35±5.32), ranging from 24 to 78 years. Clinical stages: Stage I (13 cases), stage II (21 cases), stage III (19 cases); Tumor location: descending colon (17 cases), transverse colon (16 cases), ascending colon (20 cases). There was no significant difference in the general data between 2 groups (P>0.05).

### Nursing methods

The CG received routine nursing interventions, including pain relief, hemostasis, health education, anti-inflammatory treatment, and continuous monitoring of vital signs. Additional measures, such as transfusion and fasting, were carried out according to the doctor’s instructions.

The OG received high-quality nursing interventions, which included:

1. **Environmental Nursing:** Nurses established a positive nurse-patient relationship, maintained a clean and well-ventilated ward, and ensured patient comfort.
2. **Psychological Counseling:** Nurses assessed patients’ psychological needs and provided individualised counselling. They explained the surgery process, addressed patient concerns, and shared success stories to alleviate anxiety.
3. **Health Education:** Nurses provided patients and their families with educational materials on colon cancer through lectures, videos, and manuals. They also answered questions to ensure a better understanding of the treatment process.
4. **Perioperative Nursing:**

- **Preoperative:** Nurses encouraged patients and supported them in managing anxiety, ensuring readiness for surgery.
- **Intraoperative:** Nurses closely monitored vital signs and provided protective care based on the patient’s needs and tolerance.
- **Postoperative:** Nurses monitored patients for complications, managed gastrointestinal decompression, and helped with positioning to prevent venous thrombosis. They also guided patients in turning exercises to aid recovery.

### Nutrition support methods

The CG fasted on the day of operation, and was given routine parenteral nutrition support treatment after operation, and was given fatty milk amino acid (17)/glucose (11%) injection (Product name: Caven, Fresenius Kabihuari Pharmaceutical Co., LTD., specification: 1440 mL/bag) 1440 mL/d, which was connected with deep venous tube (through internal jugular vein catheterisation) through infusion tube, and slowly dripped (at a rate of 30–45 drops /min) once a day. The recovery of the gastrointestinal tract in patients was observed. If there was no adverse reaction, such as abdominal distension, nausea, vomiting, etc., a liquid diet could be gradually opened 2 to 3 days after surgery.

The OG was supported by immune-type enteral nutrition and given Reneng Emulsion (Huarui Pharmaceutical Co. LTD, specification: 500 ml/bottle), 500 mL/d, 1 time/day; Oral administration was started 2 to 3 days before surgery, fasting on the day of surgery, oral administration again 6 to 24 hours after surgery, liquid diet 24 hours after surgery, and semi-liquid diet 48 hours after surgery until discharge.

### Observation indicators

(1) The postoperative recovery time of 2 groups was observed, including the recovery time of bowel sound, time of exhaust gas, time of defecation, time of getting out of bed and hospital stay.

(2) Nutritional indicators: Fasting peripheral venous blood was collected 1 day before surgery and 7 days after surgery in the morning, and serum transferrin (TFN), serum albumin (ALB) and prealbumin (PA) were detected by Hitachi 7600 automatic biochemical detector.

(3) Immune function indicators: Immuno globulin G (IgG), Immunoglobulin M (IgM) and immunoglobulin A (IgA) were detected by flow cytometer 1 day before surgery and 7 days after surgery.

(4) Indicators of inflammation: Interleukin (IL-6) and serum C-reactive protein (CRP) were measured by ELISA and immunoturbidimetry 1 day before surgery and 7 days after surgery, respectively.

(5) QLQ-C30 evaluated patients’ quality of life in the two groups. The QLQ-C30 score scale includes five functional areas: overall quality of life, physical function, cognitive function, role function, social function and emotional function. The specific scoring method was referred to in the reference, and the score of each item represents a better quality of life.

(6) The incidence of complications of intestinal obstruction, pulmonary infection and lower extremity deep vein thrombosis were observed and compared between 2 groups.

(7) Nursing satisfaction was evaluated by our hospital’s self-made questionnaire, including the professional level of nursing staff, service attitude, service skills, etc., with a total score of 100, divided into very satisfied (>85 points), basically satisfied (60–85 points), dissatisfied (<60 points). Total satisfaction = (very satisfied + basically satisfied)/Total cases ×100%.

### Statistical analysis

SPSS 24.0 statistical software was adopted for data analysis. Measurement data were expressed as (x̄±s), and a t-test was adopted for comparison. Count data were expressed as (n, %), and the χ^2^ test was used for comparison. P<0.05 meant statistical significance.

## Results

### Demographic and clinical characteristics

A total of 106 patients were enrolled, with 53 in each group. In the control group (CG), there were 28 males and 25 females, with an average age of 50.26±5.25 years. The clinical stages included Stage I (12 cases), Stage II (22 cases), and Stage III (19 cases). Tumour locations were distributed as follows: descending colon (16 cases), transverse colon (18 cases), and ascending colon (19 cases). In the observation group (OG), there were 30 males and 23 females, with an average age of 50.35±5.32 years. The clinical stages were Stage I (13 cases), Stage II (21 cases), and Stage III (19 cases). Tumour locations in the OG included descending colon (17 cases), transverse colon (16 cases), and ascending colon (20 cases). No significant differences in baseline characteristics were found between the two groups (P>0.05).

### Postoperative recovery time in 2 groups

**Figure 1:**
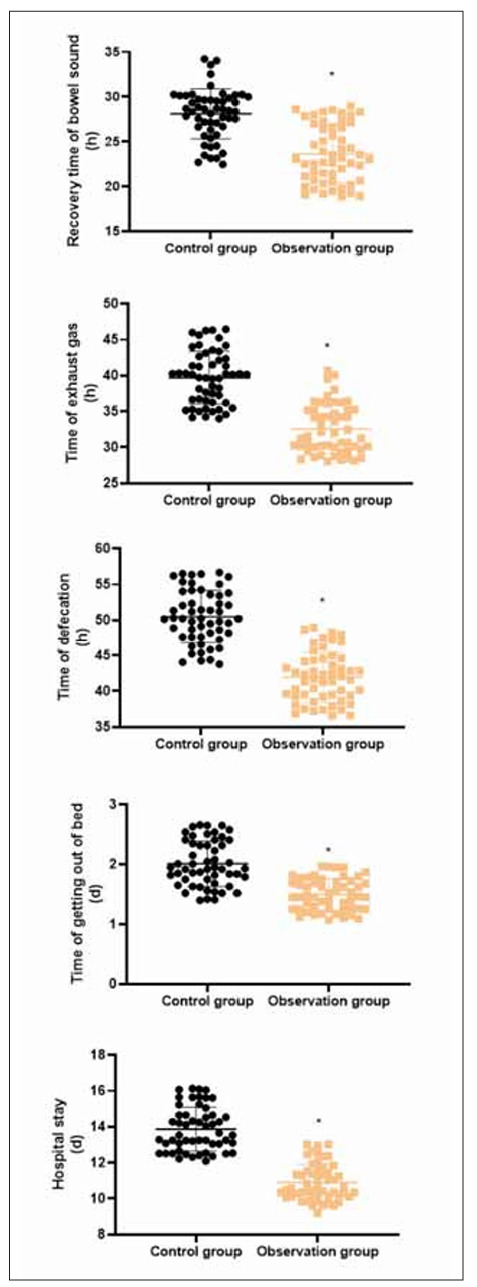
Box Plot of Hb and Alb in the 3 groups at each time point.<br>*P<0.05.

Figure 1 revealed that compared with the CG, the recovery time of bowel sound, time of exhaust gas, time of defecation, time of getting out of bed and hospital stay in the OG presented shorter (P<0.05).

### Nutritional indicators in 2 groups

**Figure 2:**
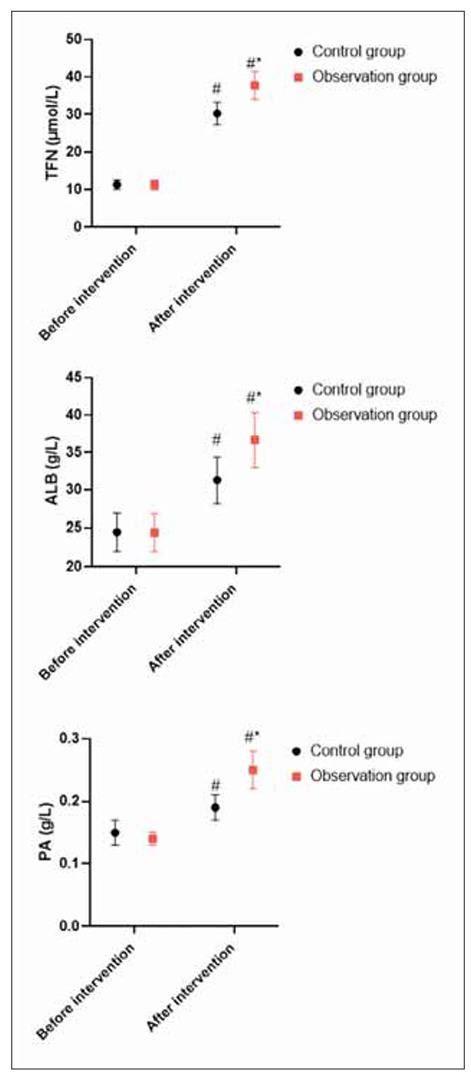
Nutritional indicators in 2 groups.<br>In contrast to before intervention, ^#^ meant P<0.05. In comparison to CG, ^*^ meant P<0.05.

Before the intervention, no difference was seen in changes in nutritional indicators between 2 groups (P>0.05). After intervention, TFN, PA and ALB levels were elevated in 2 groups, and those in the OG presented elevation when compared with the CG (P<0.05, Figure 2).

### Immune function in 2 groups

**Figure 3:**
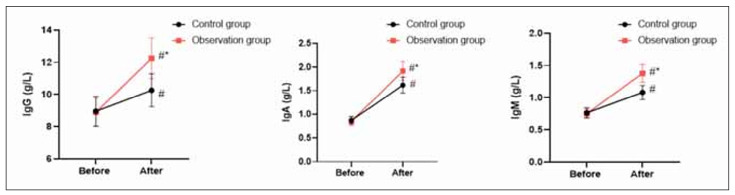
Immune function in 2 groups.<br>*P<0.05.

Before the intervention, no difference was seen in IgG, IgA and IgM levels between 2 groups (P>0.05). After the intervention, IgG, IgA and IgM levels were elevated in 2 groups, and those in the OG presented elevation when compared with the CG (P<0.05, Figure 3).

### Inflammation response in 2 groups

**Figure 4:**
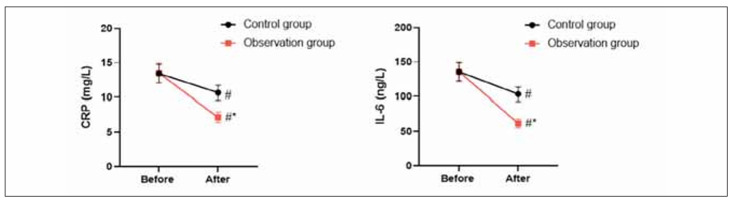
Inflammation response in 2 groups.<br>In contrast to before intervention, ^#^ meant P<0.05. In comparison to CG, ^*^ meant P<0.05.

Before the intervention, no difference was seen in changes in inflammatory factors between 2 groups (P>0.05). After the intervention, CRP and IL-6 levels declined in 2 groups, and those in the OG presented a reduction compared to the CG (P<0.05, Figure 4).

### Quality of life in 2 groups

**Figure 5:**
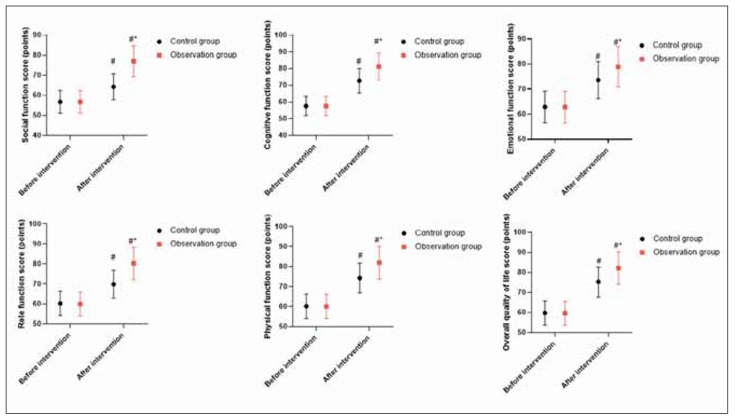
Quality of life in 2 groups.<br>In contrast to before intervention, ^#^ meant P<0.05. In comparison to CG, ^*^ meant P<0.05.

Before the intervention, no difference was seen in QLQ-C30 scores between 2 groups (P>0.05). After the intervention, QLQ-C30 scores were elevated in 2 groups, and those in the OG presented elevation when compared with the CG (P<0.05, Figure 5).

### Incidence of complications in 2 groups

Table 1 displayed that compared with the CG, the incidence of complications in the OG exhibited lower (P<0.05).

**Table 1:** Incidence of complications in 2 groups.

Groups	Cases	Intestinal obstruction	Pulmonary infection	Lower extremity deep	Total incidence rate
Control group	53	3	4	2	9 (16.98%)
Observation group	53	1	1	0	2 (3.77%)
χ^2^					4.970
P					0.025

### Nursing satisfaction in 2 groups

Table 2 displayed that compared with the CG, the nursing satisfaction in the OG exhibited better (P<0.05).

**Table 2:** Nursing satisfaction in 2 groups.

Groups	Cases	Very satisfied	Basically satisfied	Dissatisfied	Total satisfaction rate
Control group	53	23	20	10	43 (81.13%)
Observation group	53	26	25	2	51 (96.23%)
χ^2^					6.014
P					0.014

## Discussion

With the continuous development of the economy in recent years, people’s living standards continue to improve, people’s diet structure has gradually changed, and unhealthy eating habits have resulted in an increasing number of colon cancer patients [18]. This common malignant tumour of the digestive system seriously affects the daily life of patients. After the implementation of surgical resection, patients are prone to exhaust difficulties, deep vein thrombosis and other complications, which will cause patients to produce different degrees of psychological pressure, anxiety, depression and other negative emotions, which is very unfavourable to the effect of surgical treatment, as well as patients in the postoperative recovery situation [19]. Therefore, carrying out scientific and effective nursing interventions is very important.

As a clinical nursing measure widely used and generally obtains high patient satisfaction, high-quality nursing is selected as the nursing intervention in this study. Compared with conventional nursing intervention, high-quality nursing intervention mainly provides patients with a more systematic and comprehensive nursing experience through preoperative and postoperative nursing [20]. Through proactive communication and exchange before surgery, better communication can be established between nurses and patients, increase mutual trust, further enhance patients’ awareness of the operation, and reduce patients’ tension and other negative emotions, thus improving their compliance and ensuring smooth operation. Strict and close monitoring after surgery can promptly detect any abnormalities or complications in patients to prevent infections and other situations effectively. According to the actual situation of patients, corresponding rehabilitation training plans can be formulated for patients, such as rolling-over training, which can effectively promote the further recovery of patients’ gastrointestinal function after surgery.

Patients with colorectal cancer are accompanied by different degrees of malnutrition; combined with the stress response of radical surgery, the body of patients is in a state of high decomposition and large energy consumption, resulting in insufficient nutrient supply of intestinal mucosal cells and damage to their barrier function [21]. Abnormal intestinal mucosal barrier function is easy to cause bacterial translocation, triggers inflammatory reactions of intestinal epithelial cells, and affects the outcome and rehabilitation of patients with disease [22]. Therefore, giving patients reasonable nutritional support treatment after surgery is imperative.

Parenteral nutrition is an economical, effective, and safe nutritional support method widely used in clinical practice [23]. Although the conventional nutrient solution can promote the absorption of gastrointestinal nutrition and improve the state of malnutrition, inflammation, and immunosuppression in patients to a certain extent, the improvement effect is not good [24]. Studies have shown that, compared with conventional nutritional solutions, immune-type enteral nutrition support can effectively promote postoperative recovery of patients and improve patient prognosis [25].

The immune-type nutrient solution is rich in special nutrients such as glutamine, arginine and omega-3 unsaturated fatty acids [26]. Glutamine is a kind of free amino acid with the highest content in the human body and is closely related to the metabolism of gastrointestinal epithelial cells, lymphocytes, and macrophages [27]. When the body is stimulated by trauma, infection and other stimuli in a strong stress state, the level of glutamine in the human body will decrease significantly [28]. Timely supplementation of glutamine in patients undergoing radical gastrectomy can help protect the structural integrity of intestinal mucosa, maintain its normal metabolic function, and enhance the intestinal immune function of patients [29]. Arginine is a semi-essential amino acid in the human body, and the supplementation of arginine for patients in a state of stress due to trauma and infection is beneficial to synthesising protein and regulating immune function [30]. ω-3 unsaturated fatty acids can effectively reduce inflammatory factors such as IL-1, IL-6 and TNF-α in patients, reduce the body’s inflammatory response and improve immunity [31].

In our study, the results revealed that in comparison with the CG, the recovery time of bowel sound, time of exhaust gas, time of defecation, time of getting out of bed and hospital stay in the OG presented shorter, and the incidence of complications in the OG exhibited lower. All these findings suggested that high-quality nursing combined with immune-type enteral nutrition support could accelerate postoperative recovery and reduce the incidence of complications in patients undergoing radical resection of colon cancer. Shi et al. consistently indicated that high-quality nursing for patients undergoing brain tumour surgery can reduce the complication rate [32]. Jiang et al. have suggested that immune-enhanced enteral nutrition can shorten patients’ hospital stay after an operation [33].

Besides, our study indicated that after intervention, in comparison with the CG, TFN, PA and ALB levels in the OG presented elevation, IgG, IgA, and IgM levels in the OG presented elevation, and CRP and IL-6 levels in the OG presented reduction. All these results suggested that high-quality nursing combined with immune-type enteral nutrition support could promote the nutritional status and immune function along with reducing the inflammatory response of patients undergoing radical resection of colon cancer, which was similar to studies proposed by Chen et al., Hirofumi Shirakawa et al. and Fang et al. [34] [35] [36]. Shirakawa and colleagues investigated the effects of preoperative enteral immunonutrition with an immune-enhanced formula (Impact) in patients undergoing pancreaticoduodenectomy. The study found that the formula was well tolerated and resulted in fewer wound infections and a milder systemic stress response than the control group, highlighting its potential benefits in improving postoperative recovery.

Moreover, our study indicated that after the intervention, in comparison with the CG, the QLQ-C30 scores and nursing satisfaction in the OG exhibited better, suggesting that high-quality nursing combined with immune-type enteral nutrition support could promote the quality of life and nursing satisfaction of patients undergoing radical resection of colon cancer, which was consistent with previous studies [37] [38].

In conclusion, our study clarifies that high-quality nursing combined with immune-type enteral nutrition support can promote postoperative recovery, enhance nutritional status and immune function, and reduce complications in patients undergoing radical resection of colon cancer.

## Conclusion

In conclusion, the combination of high-quality nursing interventions and immune-type enteral nutrition support significantly enhances the postoperative recovery of patients undergoing radical resection of colon cancer. Our study demonstrated that this approach accelerates the recovery process, improves nutritional status, boosts immune function, and reduces the incidence of complications compared to conventional care. Additionally, it contributes to better quality of life and higher patient satisfaction. These findings highlight the importance of integrating comprehensive nursing strategies with advanced nutritional support to optimise colon cancer patients’ recovery outcomes and overall well-being post-surgery.

## Dodatak

### Conflict of interest statement

All the authors declare that they have no conflict of interest in this work.
